# Sanitation in the West Indies

**Published:** 1909-07-03

**Authors:** Ronald Ross


### EDITOR'S LETTER-BOX.

### SANITATION IN THE WEST INDIES.

*To the Editor of* The Hospital.

The Incorporated Liverpool School of Tropical Medicine,
Johnston Tropical Laboratory, University of Liverpool.

Sir,—In your issue of June 19 you contrast the statements of Sir Rubert Boyce and myself on this matter; but I should like to point out that my strictures refer to the past ten years, while Professor Boyce's experiences refer only to the last few months. Moreover, my strictures were of a general nature and based, regarding the West Indies at least, on information supplied to me by others, and especially on certain defective reports. Sir Rubert Boyce, being rather new to this business, is probably more optimistic than an old hand like myself. I sincerely hope, however, that now, as a result chiefly of his own active interest in the matter, something worthy of the name of antimalaria campaign will be done in the colonies you refer to. I may add that an expedition of ours which has just returned from Jamaica (which was not visited by Professor Boyce) did not describe any very extensive anti-mosquito campaigns undertaken there.

Yours faithfully,

June 23, 1909. Ronald Ross.

